# Autistic children’s language imitation shows reduced sensitivity to ostracism

**DOI:** 10.1007/s10803-021-05041-5

**Published:** 2021-06-08

**Authors:** Zoë L. Hopkins, Nicola Yuill, Holly P. Branigan

**Affiliations:** 1 Department of Psychology, University of Edinburgh, 7 George Square, Edinburgh, EH8 9JZ UK; 2 School of Psychology, University of Sussex, Falmer, Brighton, BN1 9QH UK

**Keywords:** Autism, Affiliation, Alignment, Conversation, Language imitation, Ostracism

## Abstract

In dialogue, speakers tend to imitate, or align with, a partner’s language choices. Higher levels of alignment facilitate communication and can be elicited by affiliation goals. Since autistic children have interaction and communication impairments, we investigated whether a failure to display affiliative language imitation contributes to their conversational difficulties. We measured autistic children’s lexical alignment with a partner, following an ostracism manipulation which induces affiliative motivation in typical adults and children. While autistic children demonstrated lexical alignment, we observed no affiliative influence on ostracised children’s tendency to align, relative to controls. Our results suggest that increased language imitation—a potentially valuable form of social adaptation—is unavailable to autistic children, which may reflect their impaired affective understanding.

## Introduction

Autism is characterised by deficits in social communication and social interaction (DSM-5; American Psychiatric Association, 2013), which can manifest in conversational language (Ying Sng et al., 2018). In typical dialogue, linguistic imitation between conversational partners—or *alignment*—is a common feature (cf. Pickering & Garrod, 2004), and higher levels of alignment are associated with effective, rewarding exchanges between speakers (Fusaroli et al., 2012; Putman & Street, 1984). Although alignment has been considered primarily as arising from automatic priming and cue-based memory (Horton & Gerrig, 2005; Pickering & Garrod, 2004) and/or audience design mechanisms (Branigan et al., 2011; Brennan & Clark, 1996), speakers also align for social-affective reasons (Giles et al., 1991). Since autism is associated with social-affective difficulties, including reduced social orientation and affiliative behaviour (Chevallier, Grèzes, et al., 2012; Chevallier, Kohls, et al., 2012; Klin, 1991), we might expect that social-affective factors would not influence autistic people’s alignment to a typical extent. Here we examine whether verbal autistic children align atypically in response to an experience of ostracism, in ways that could undermine their conversational ability.

Communication and interaction deficits in autism are commonly viewed as resulting from impaired understanding of others’ thoughts, beliefs, and intentions, or ‘theory of mind’ (Baron-Cohen et al., 1985). This impairment involves cognitive deficits in meta-representational capacity (Baron-Cohen, 1988), as well as problems with social-affective relatedness (Dawson & Lewy, 1989; Mundy & Sigman, 1989). Hobson (1989, 1993) has argued that autistic individuals lack the capacity to recognise and respond to the emotional states of others, which limits their development of interpersonal connections and thus their social understanding. In more recent work, conversational difficulties in autism have been directly linked with the failure to identify with a social partner on an affective level. For example, autistic children who show signs of reduced affiliative behaviour (e.g. smiling)—relative to non-autistic children—are less able to infer their interlocutor’s intended meanings (Hobson et al., 2012), and tend to engage in less co-ordinated conversation (García-Pérez et al., 2007). Furthermore, there is evidence that autistic children’s emotional connectedness with a partner correlates with their use of first person plural pronouns (Hobson et al., 2010), raising the possibility that the affective deficits associated with autism might manifest in language. However, we know of no work that examines how affective factors influence autistic children’s alignment in conversation. Since alignment contributes to effective, rewarding communication, this issue is important for understanding how and under what circumstances conversational difficulties might arise in autism.

We base our investigation on the ‘perception-behaviour’ link identified in studies of non-linguistic imitation, which have revealed a bidirectional relationship between imitation and affiliative behaviour in the typical population (Dijksterhuis & Bargh, 2001). This link is observable in very young typically-developing children’s behaviour: For example, being mimicked by an experimenter promotes a pro-social orientation towards others in eighteen-month-olds (Carpenter et al., 2013). In adulthood, people experience increased liking of partners who mimic their posture and movements (Chartrand & Bargh, 1999), and tend to mimic a partner they like more than one they do not (Stel et al., 2010). These effects extend to conversational alignment: speakers who converge on a partner’s vocabulary range are evaluated more favorably than those who do not (Bradac et al., 1988), and such positive affect may generate tangible benefits for the mimicker (van Baaren et al., 2003).

Further evidence for a link between imitation and affiliation comes from studies which have experimentally manipulated social exclusion (specifically, ostracism) and compared the behaviour of participants who experienced ostracism with controls who experienced inclusion. For example, typical adults who are ostracised are more likely than included controls to mimic a social partner’s physical mannerisms (e.g., Lakin et al., 2008). Similarly, young typically-developing children exposed to ostracism imitate an experimenter’s actions more closely than included controls (Over & Carpenter, 2009), a trend which is amplified when imitating social-conventional rather than instrumental actions (Watson-Jones et al., 2014), and when exclusion is enacted by in-group rather than out-group members (Watson-Jones et al., 2016). A recent study applied this experimental approach to typically-developing children’s alignment of word choice (i.e., lexical alignment), and found that those who experienced ostracism in a virtual ball-tossing game displayed increased lexical alignment with a partner, compared to included controls (Hopkins & Branigan, 2020). The effect of ostracism on imitation has been primarily explained in terms of goal activation theory (cf. Aarts & Dijksterhuis, 2000). According to this theory, the goal to affiliate is directly activated when we feel our sense of belonging is under threat; in order to fulfil these affiliative goals, we consequently display more behaviours that communicate our similarity to a social partner (Over & Carpenter, 2009).

While imitation seems to be sensitive to ostracism in the typical population, converging lines of evidence suggest that such effects might not manifest in autism. Firstly, studies of non-linguistic imitation in autistic children and adults report a deficit when the to-be-copied behaviour involves a social component (Wang & Hamilton, 2012; see also Vivanti & Hamilton, 2014, for a review). For example, autistic children are less likely than typically-developing children to over-imitate an adult’s unnecessary actions (Marsh et al., 2013; though cf. Nielsen et al., 2013), and their action imitation is not modulated by social contextual cues, compared with non-autistic controls (Vivanti & Dissanayake, 2014) and children with Williams Syndrome (Vivanti et al., 2016). Importantly, it has been shown that priming autistic adults with a pro-social attitude does not lead to increased levels of non-linguistic imitation (compare to those primed with a non-social attitude), as it does in typical controls (Cook & Bird, 2012).

Secondly, there is evidence for atypical processing of ostracism in autism: Autistic individuals report typical levels of distress in response to ostracism, but display relative hypoactivity in the neural circuitry that deals with rejection (Bolling et al., 2011; Masten et al., 2011; McPartland et al., 2011; Sebastian et al., 2009). Furthermore, although autistic adults experience a heightened physiological response to ostracism, they do not interpret ostracism as emotionally significant to the same degree as non-clinical controls (Trimmer et al., 2017). Such findings may reflect the influence of alexithymic traits—difficulties with recognising and describing one’s own emotional states—which are prevalent in the autistic population (50% compared with 10% in the typical population; Bernhardt et al., 2014; Hill et al., 2004), and which are associated with language impairment (Hobson et al., 2019; Milosavljevic et al., 2016). Taken together, the findings from these two literatures raise the possibility that autistic individuals’ imitative behaviour might not be modulated by ostracism.

Hence in the current study, we predict that autistic children who experience ostracism will not follow the pattern of typically-developing children of increased lexical alignment relative to included controls (Hopkins & Branigan, 2020). Previous studies have found that priming mechanisms giving rise to alignment are intact for autistic children, such that they align to the same extent as groups of typically-developing children matched by either age or verbal ability (Allen et al., 2011; Branigan, et al., 2016; Hopkins, Yuill, & Branigan, 2017; Hopkins, Yuill, & Keller, 2016). But these results were all found in contexts where social-affective factors were not central to the interaction. They therefore do not elucidate whether social-affective factors might modulate alignment. Here we set out to tap the social-affective mechanisms of autistic children’s alignment, by manipulating their inclusionary status before they interact with a conversational partner. If autistic children show an atypical pattern of alignment in response to ostracism, this might partly explain why their conversational behaviour appears unusual. Moreover, given the bi-directional link between imitation and affiliation, atypical alignment could also help to explain why partners might perceive conversation with autistic children as dissatisfying or unrewarding.

The current study adopted the same protocol reported by Hopkins and Branigan (2020). Autistic children experienced either ostracism or inclusion via the Cyberball paradigm (Williams et al., 2000), before playing a picture-naming game with an experimenter, based on the children’s card game ‘snap!’ (Branigan et al., 2016). In the game, each card depicted a familiar object that had two acceptable names (e.g., rabbit vs. bunny). On experimental trials, the child heard the experimenter name her picture with either a *favored* name (rabbit; as established by a pre-test) or a *disfavored* alternative (bunny). Two turns later, the child named the same object. Children’s tendency to imitate the experimenter’s use of disfavored names was recorded, and the extent of lexical alignment was compared between the ostracism and control (i.e., inclusion) groups. If autistic children’s priming-based tendency to align were further strengthened by the concurrent action of social-affective mechanisms, then those who experienced ostracism should lexically align with the experimenter to a greater extent than controls, consistent with typically-developing children (Hopkins & Branigan, 2020). However, a lack of difference in lexical alignment between the ostracised and included groups would be consistent with an impairment of social-affective mechanisms of alignment in autistic children.

## Method

### Participants

Participants were 23 children (17 male; mean age [in years; months] = 9;8; age range = 6;8–11;6) attending special educational needs (SEN) schools (*N* = 10) and SEN units within mainstream primary schools (*N* = 13) in Dorset and Sussex, UK. Although a larger sample would have been desirable, autistic children are a difficult-to-reach population, and we note that our sample size compares favorably with other investigations of autistic children’s lexical alignment (e.g., *N* = 15 in Branigan et al., 2016; *Ns* = 12 and 14 in Hopkins et al., 2017). All children had received a formal diagnosis of autism as part of a detailed, multi-disciplinary assessment led by a psychiatrist or clinical psychologist; formal diagnosis was a pre-condition for admission to the SEN settings attended by the children. As such, in line with Ambridge et al. (2020) and consistent with gold-standard UK prevalence research on autism (e.g., Baron-Cohen et al., 2009), we assumed that false positives in diagnosis were non-existent, and did not subject children to extensive re-evaluation of their autistic symptoms. Instead, we assessed each child’s social-communicative functioning via the current version of the Social Communication Questionnaire (SCQ; Rutter et al., 2003); we used scores on this parent-report screening tool as a proxy for children’s autism severity.

Children were randomly assigned to either the ostracism or control group for Cyberball, and the groups were well matched for chronological age, receptive and expressive vocabulary (assessed via the verbal scale of the Kaufman Brief Intelligence Test—Second Edition; Kaufman & Kaufman, 2004), SCQ scores, and gender (see Table 1).

**Table 1:** Participant characteristics (ages in years;months) by group

	Group		
	Control	Ostracism	*p* value
Chronological age	*M* = 10;0 (range 8;1–11;6)	*M* = 9;5 (range 6;8–10;10)	.24^b^
Receptive vocabulary^a^	*M* = 27.0 (*SD* = 11.27)	*M* = 25.33 (*SD* = 6.84)	.67^b^
Expressive vocabulary^a^	*M* = 21.27 (*SD* = 9.39)	*M* = 20.67 (*SD* = 6.72)	.86^b^
SCQ	*M* = 16.27 (*SD* = 5.16)	*M* = 14.17 (*SD* = 4.41)	.30^b^
Gender (M:F)	8:3	9:3	.90^c^

^a^Raw score

^b^No significant group difference on an independent *t*-test

^c^No significant group difference on a Chi-square test

### Materials

All children were administered the following tasks in a fixed order: Cyberball (either ostracism or control)—> snap! game—> verbal scale of the Kaufmann Brief Intelligence Test (KBIT-2)—> Cyberball (inclusion only).

#### Cyberball (Williams et al., 2000)

Cyberball is a computerised game, in which a participant throws a ball back and forth with two pre-programmed confederates. We followed Zadro et al.’s (2013) recommendations in adapting Cyberball for children, who played the game on a laptop. Initially, children saw a screen that explained how to play Cyberball, and that also—to deflect attention away from the manipulation—instructed them to use their imagination during the game. The School of Philosophy, Psychology, & Language Sciences Research Ethics Committee (PPLSREC) at the University of Edinburgh approved the use of Cyberball in our study (IRB Protocol Number: 207–1617/2).

**Fig. 1:**
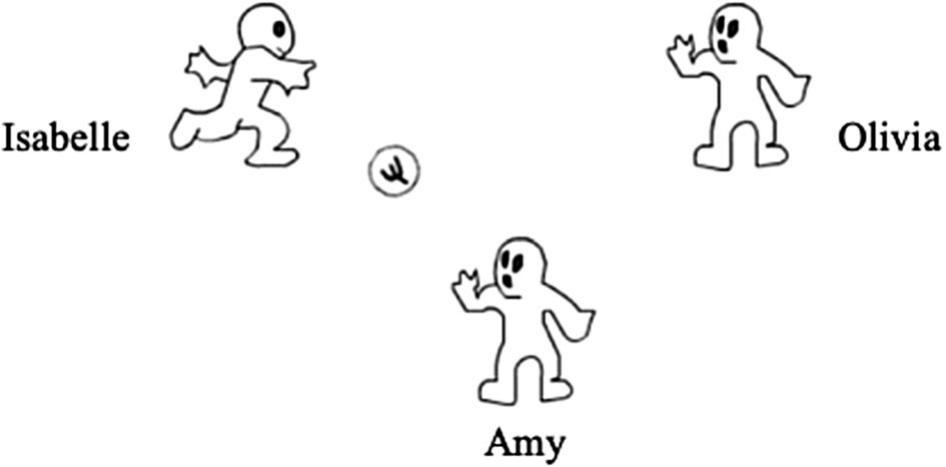
Screenshot of Cyberball game

On the laptop screen, each child was represented by an animated avatar bearing his or her name (Fig. 1); the names for confederate avatars were randomly drawn from lists of popular boys’ and girls’ names in England and Wales (Office for National Statistics, 2015). Blind-coding of game syntax kept the experimenter unaware of whether a child would experience ostracism or inclusion in the experimental round of the game.

Following a practice (inclusion) round of Cyberball, children progressed to the experimental round which consisted of 20 ball-toss trials. Those in the control group received the ball from the confederates with equal probability across the 20 trials; those in the ostracism group received the ball with equal probability across the first 6 trials; thereafter, the confederates threw the ball only between themselves. While comparing ostracism and active inclusion could in principle exaggerate the effects of Cyberball, recent research suggests that more neutral control conditions provide a similar experience to inclusion in the game (Dvir et al., 2018).

#### Cyberball manipulation check

To verify that children were aware of their inclusionary status in Cyberball, we administered a manipulation check after the experimental round of the game. Children were asked ‘How much did they throw you the ball?’ and their responses were recorded via a five-point response scale where 1 = not at all and 5 = a lot (see Abrams et al., 2011). Children recorded their responses on paper forms that they privately posted into a ballot box, to maintain the experimenter’s blindness to group assignment.

#### Snap! game

We used Branigan et al.’s (2016) game, which includes 20 experimental items. Each item comprised a pair of picture cards (a *prime* and a *target*) depicting an object that had two acceptable names (e.g., rabbit; bunny), and a scripted prime name (favored vs. disfavored).

**Fig. 2:**
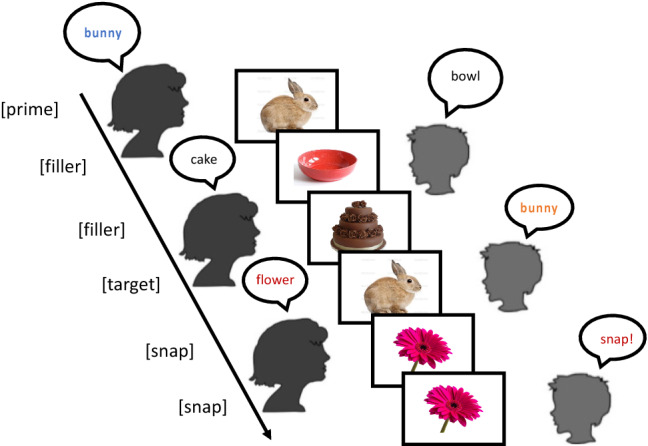
Sample experimental trial. The experimenter named an object using the favored name (“rabbit”) or disfavored name (“bunny”); after two fillers, the child named the same object. Alignment occurred if the child used the same name as the experimenter previously used (“bunny”). On snap! trials, the experimenter and child consecutively named the same object

There were two experimenter/participant lists, each containing one version of each experimental item in a Latin Square design, plus 28 filler cards (see Fig. 2). Item order was individually randomized for each child, with the constraints that (1) two fillers intervened between the experimenter’s prime and the child’s associated target, and (2) the eight ‘snap’ trials (involving adjacent pairs of identical cards) were distributed evenly throughout the game Children were randomly assigned to one of the experimenter/participant lists.

#### Language measures

The verbal scale of the KBIT-2 (Kaufmann & Kaufman, 2004) comprises two subtests: Verbal Knowledge and Riddles. The Verbal Knowledge subtest measures receptive vocabulary: It required the child to select from six pictures the one matching a name spoken by an examiner. The Riddles subtest required the child to say a word that answers riddles spoken by an examiner (e.g., *what is something shiny and hard that you wear on your finger*?).

### Procedure

Children were tested individually by the experimenter. At the start of the session, each child was given an overview of the tasks they would be asked to complete; we aimed for children to progress immediately from Cyberball to playing the snap! game, to maximise the influence of the affiliation manipulation on their subsequent behaviour in dialogue. Once children had read the instructions for Cyberball and played a practice round, the experimenter positioned herself away from the laptop screen to avoid becoming aware of the child’s group assignment in the experimental round of the game. Children then completed the Cyberball manipulation check, before playing the snap! game with the experimenter. The experimenter and child each had a pile of cards, and they took turns turning over and naming the top card. When adjacent cards were identical, the first player to say ‘snap!’ won the cards on the table. Following the snap! game, children were tested on the KBIT-2, before playing a final inclusionary round of Cyberball. This round was intended to alleviate any residual sense of ostracism that children might have felt while playing the experimental round of the game (e.g., Ruggieri et al., 2013). At the end of the session, the experimenter explained the Cyberball deception to children, both verbally and via a written information sheet.

### Coding and analysis

We classified all target responses as *Favored*, *Disfavored*, or *Other* (Table 2). We excluded *N* = 8 responses from data contributed by children who misordered some of their cards during the snap! game.

**Table 2:** Frequency (and %) of children’s target lexical responses, by prime name and group

Prime name				
Group	Response	Favored	Disfavored	Alignment effect^a^ (95% bootstrapped CIs)
Control	Favored	90 (77%)	38 (35%)	
	Disfavored	8 (7%)	64 (58%)	51% (28–67)
	Other	9	8	
Ostracism	Favored	91 (78%)	36 (30%)	
	Disfavored	13 (11%)	73 (62%)	51% (37–65)
	Other	12	9	

^**a**^Alignment effects represent percentage point increases in the observed probability of producing a disfavored response after a favored vs. after a disfavored prime name

We began our analyses by comparing the post-manipulation check scores of the ostracism and control groups, to determine that the Cyberball manipulation had been effective. We then analyzed our snap! game data using logit mixed effect models (LMEMs), which estimated the likelihood of aligning with the experimenter on a disfavored name (disfavored responses = 1; all other responses (favored/other) = 0) from the fixed effects of prime type (favored vs. disfavored), group (control vs. ostracism) and a prime type x group interaction term; critically, the interaction term would indicate whether children’s responses to the experimenter’s prime names (i.e., their alignment) varied by group. We also included language and SCQ scores as fixed effects in this analysis, and random intercepts for participant and item, which were the maximal random effect structures supported by the data. The LMEMs were fitted using the lme4 package (version 1.1–12; Bates et al., 2016) in R (version 3.3.1; R Core Team, 2019). Lastly, we used our LMEMs to calculate Bayesian Information Criteria (BIC) values, as an alternative to classical hypothesis testing. We have taken this approach in previous work (Hopkins & Branigan, 2020) when—owing to a lack of prior studies examining the influence of ostracism on alignment—it has not been possible to determine sample size via traditional power analysis; power analyses require published effect sizes to establish the threshold beneath which a hypothesis would be rendered false. Instead, Bayes Factors quantify the strength of evidence for an alternative versus null hypothesis based on our own data (Dienes, 2014; see also Masson, 2011).

## Results

### Cyberball manipulation check scores

The results of a Mann–Whitney test suggested that the Cyberball manipulation was effective. In response to the question ‘How much did they throw you the ball?’ (1 = not at all; 5 = a lot), children in the ostracism group reported receiving the ball less frequently (*Mdn* = 2) than did controls (*Mdn* = 4), *U* = 1, *p* < 0.001.

### LMEMs for snap! game data

**Fig. 3:**
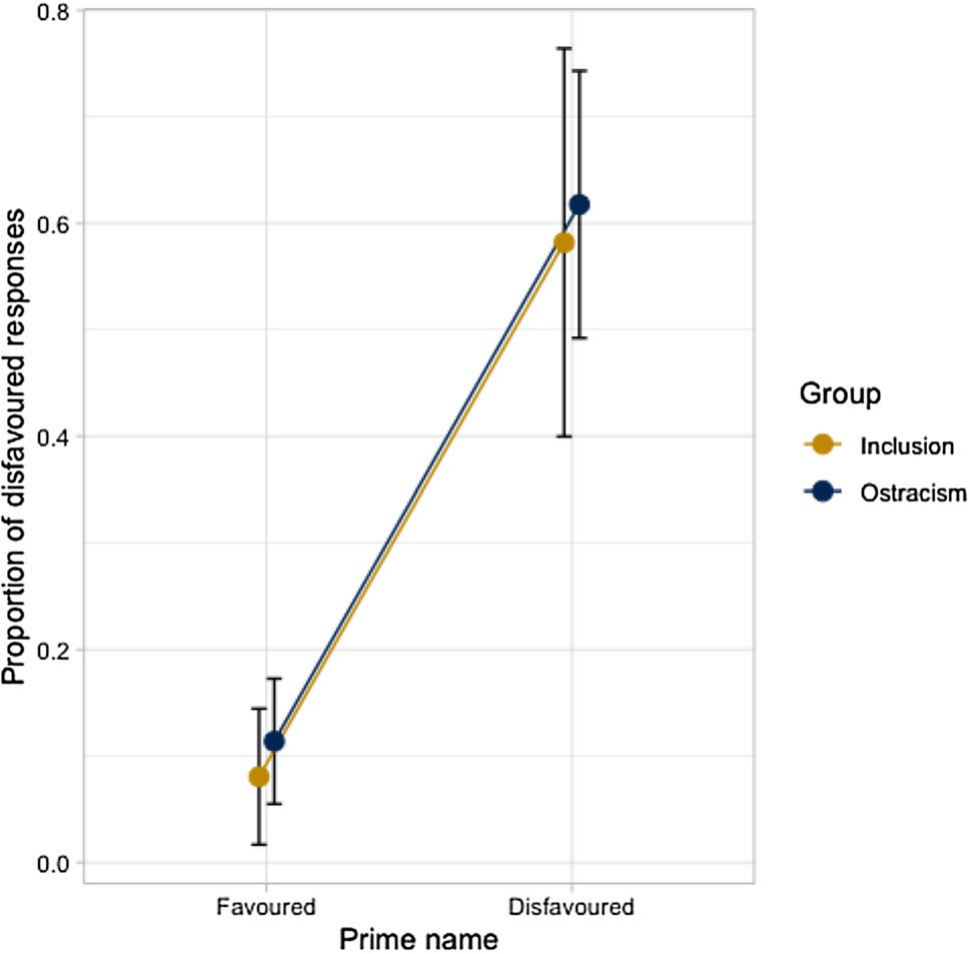
Line graph of prime name x group interaction (error bars are 95% confidence intervals)

Initially, we fitted an LMEM to the full snap! game data set, which included all fixed effects and relevant interactions to estimate children’s likelihood of aligning with the experimenter on a disfavored name. This revealed a significant effect of prime name, meaning that, overall, children were more likely to produce a disfavored name in the snap! game when they had heard the experimenter use a disfavored rather than favored name to describe the same object (60% vs. 9% disfavored responses; Tables 2 and 3). Hence children engaged in lexical alignment with the experimenter. Critically, there was no significant interaction between prime name and group, meaning that the extent of alignment did not differ between ostracised and control children (51% vs. 51% alignment effect; Table 2, Fig. 3).

**Table 3:** Summary of LME model for the likelihood of aligning on a disfavored name

	Parameter estimates	Wald’s test		
	β	S.E	Z	*p*(β = 0)
Intercept	− 1.20	0.29	− 4.18	
Prime name^a^	− 1.60	0.20	− 7.99	< .001
Group^a^	− 0.48	0.38	− 1.26	> .1
Receptive vocabulary^b^	− 0.06	0.04	− 1.38	> .1
Expressive vocabulary^b^	0.07	0.05	1.42	> .1
SCQ score^b^	0.01	0.04	0.39	> .1
Prime name:Group	− 0.49	0.37	− 1.32	> .1
Prime name:Receptive vocabulary	− 0.05	0.04	− 1.18	> .1
Prime name:Expressive vocabulary	0.02	0.05	0.46	> .1
Prime name:SCQ	0.09	0.04	2.41	< .05
Group: Receptive vocabulary	− 0.05	0.09	− 0.53	> .1
Group: Expressive vocabulary	0.09	0.09	0.92	> .1
Group: SCQ	− 0.00	0.07	− 0.04	> .1
Prime name:Group:Receptive vocabulary	− 0.20	0.09	− 2.22	< .05
Prime name:Group:Expressive vocabulary	0.11	0.09	1.20	> .1
Prime name:Group:SCQ	− 0.14	0.07	− 2.01	< .05

^a^Prime name was deviation-contrast coded, with values .5/.5 for levels favored/disfavored

^b^Receptive vocabulary, expressive vocabulary, and SCQ were all centered and scaled

**Fig. 4:**
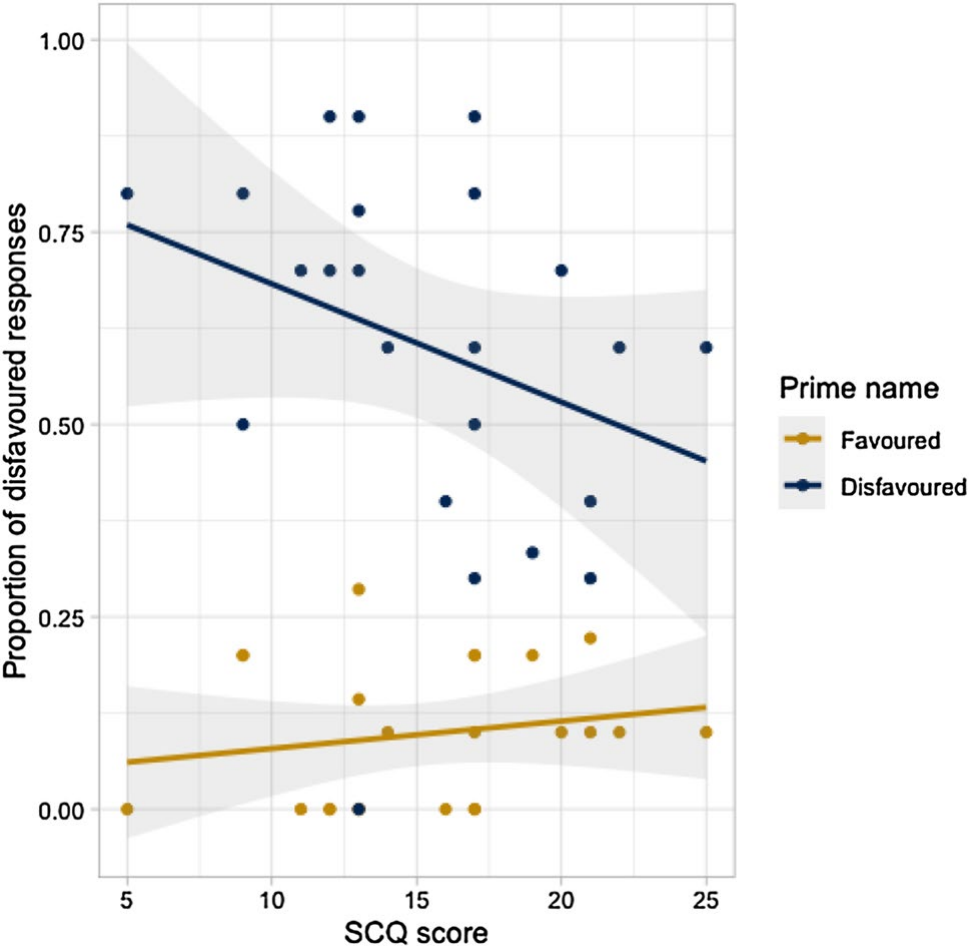
Scatterplot of interaction between alignment effects and SCQ scores (grey bands are standard error bands)

However, the LMEM revealed significant interactions between other predictors of alignment. There was a significant two-way interaction between prime name and SCQ score. This interaction is illustrated in Fig. 4, which plots SCQ scores against children’s tendency to produce a disfavored name after a favored vs. disfavored prime (i.e., alignment effects). The interaction indicated that, irrespective of group, children with higher SCQ scores (i.e., greater autistic symptom severity) tended to show weaker lexical alignment effects; that is, they were less likely to produce a disfavored name after hearing the experimenter use a disfavored name, and more likely to produce a disfavored name after hearing a favored name used.

**Fig. 5:**
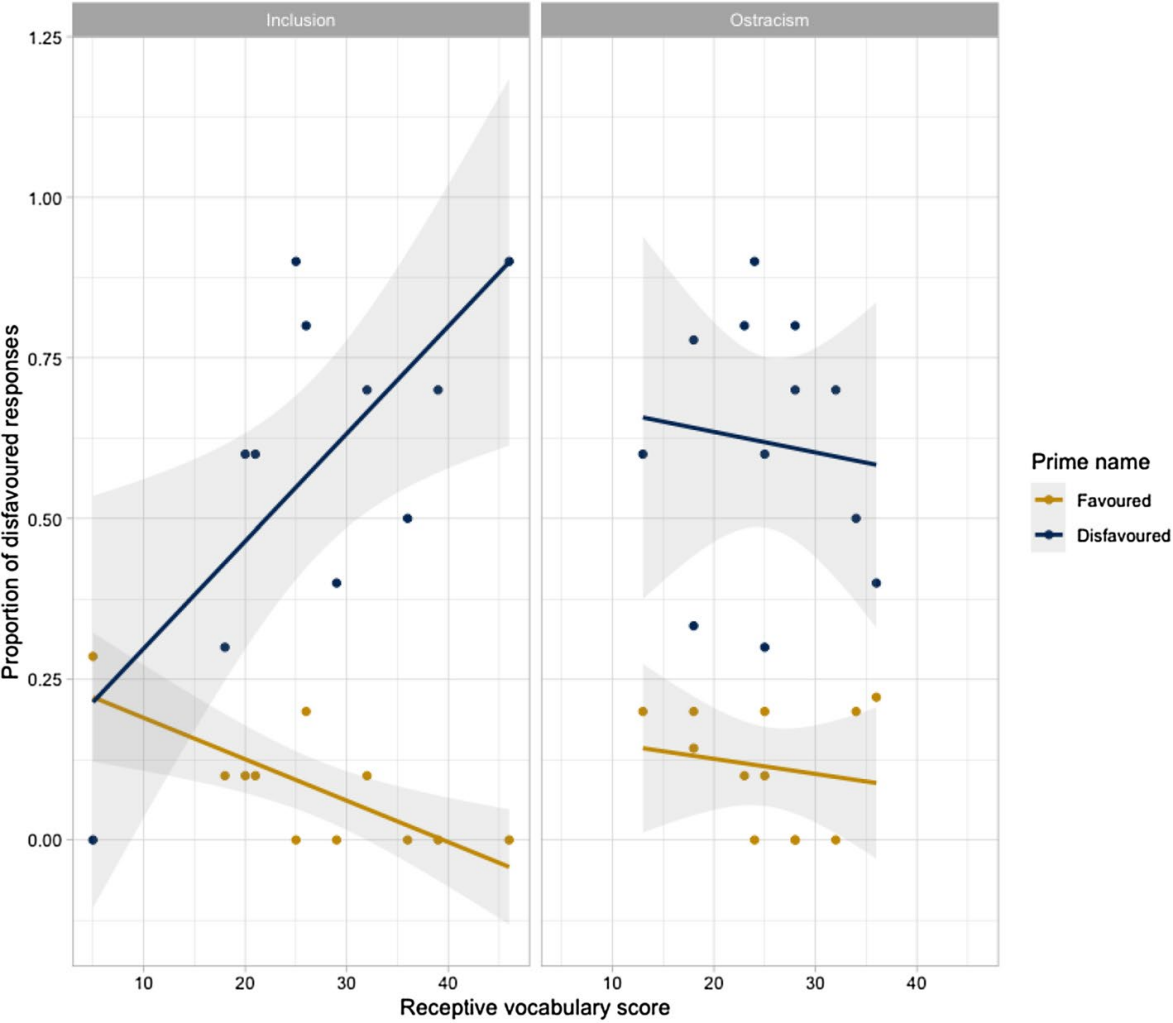
Scatterplots of interaction between alignment effects and receptive vocabulary scores, by group (grey bands are standard error bands)

Furthermore, there were significant three-way interactions between prime name, group, and receptive vocabulary, and also between prime name, group, and SCQ score. We interrogated these interactions by fitting group-specific LMEMs to the snap! game data, to investigate the relationship between (i.) receptive vocabulary and (ii.) SCQ scores in the ostracism and control groups separately. In the control group, there was a relationship between receptive vocabulary scores and alignment effects: Control children with better receptive vocabularies tended to show stronger alignment effects; they were more likely to produce a disfavored name after hearing the experimenter use a disfavored name, and less likely to produce a disfavored name after hearing a favored name. In contrast, this relationship was not apparent in the ostracism group (Table 4; Fig. 5).

**Table 4:** Summary of LME models for the likelihood of aligning on a disfavored name, by group

		Parameter estimates		Wald’s test	
Group	Fixed effects	β	S.E	Z	*p* (β = 0)
Control	Intercept	− 1.68	0.52	− 3.26	
	Prime name^a^	− 4.30	0.85	− 5.07	< .001
	Receptive vocabulary^b^	− 1.29	.99	− 1.31	> .1
	Expressive vocabulary^b^	1.23	0.89	1.38	> .1
	SCQ score^b^	0.13	0.35	0.37	> .1
	Prime name:Receptive vocabulary	− 4.13	1.80	− 2.29	< .05
	Prime name:Expressive vocabulary	1.84	1.46	1.26	> .1
	Prime name:SCQ	0.29	0.54	0.53	> .1
Ostracism	Intercept	− 0.93	0.27	− 3.39	
	Prime name	− 3.02	0.44	− 6.84	< .001
	Receptive vocabulary	− 0.24	0.37	− 0.66	> .1
	Expressive vocabulary	0.14	0.36	0.40	> .1
	SCQ score	0.05	0.22	0.24	> .1
	Prime name*receptive vocabulary	0.59	0.75	0.79	> .1
	Prime name:Expressive vocabulary	− 0.41	0.71	− 0.58	> .1
	Prime name:SCQ	1.35	0.46	2.92	< .01

^a^Prime name was deviation-contrast coded, with values .5/.5 for levels favored/disfavored

^b^Receptive vocabulary, expressive vocabulary, and SCQ were all centered and scaled

**Fig. 6:**
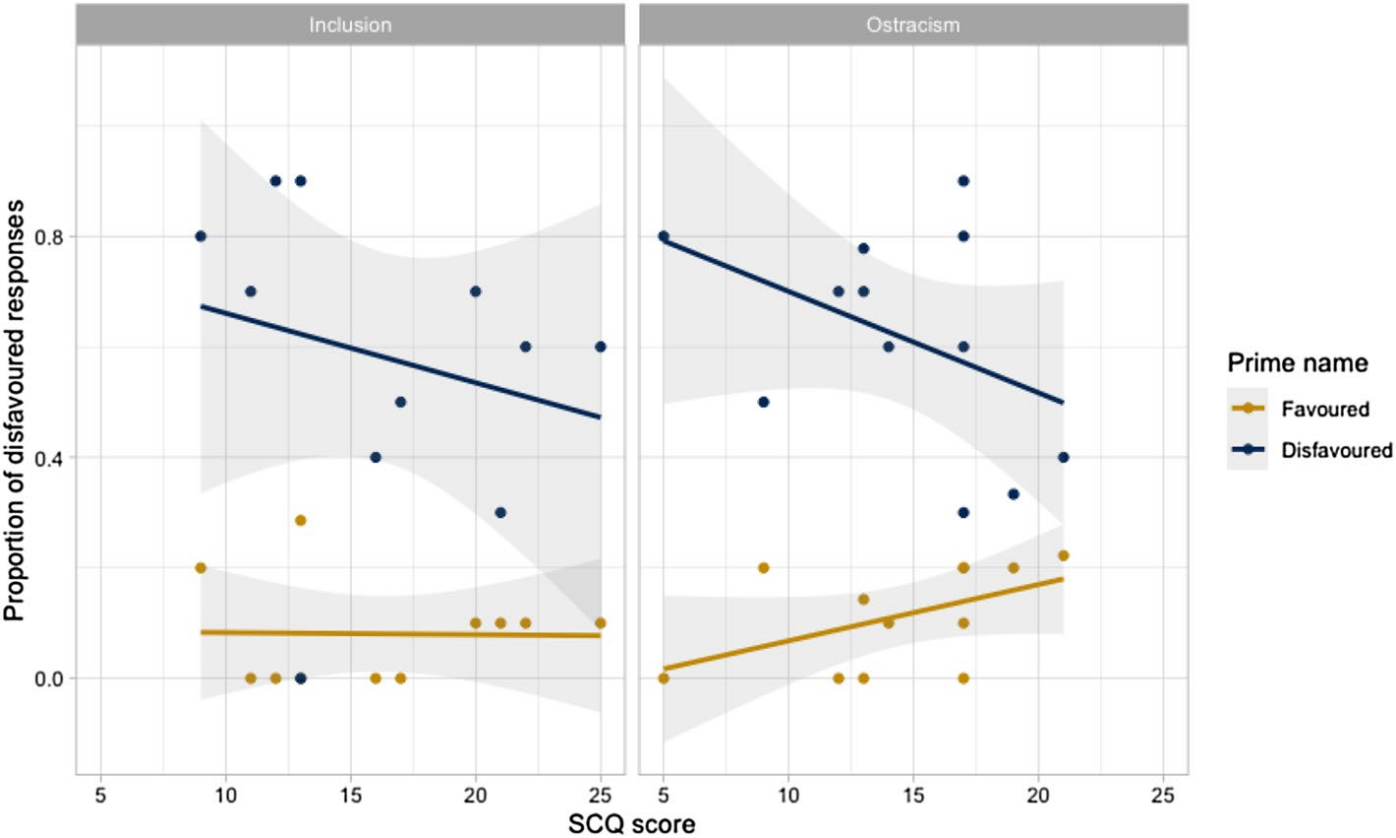
Scatterplots of interaction between alignment effects and SCQ scores, by group (grey bands are standard error bands)

The group-specific LMEMs also clarified that the significant two-way interaction between prime name and SCQ in the full LMEM was driven by the behavior of children in the ostracism group. Although children with higher SCQ scores tended to show numerically weaker alignment effects in both the ostracism and control groups, the relationship between these fixed effects was significant for the ostracism group only (Table 4; Fig. 6).

Taken together, these results suggest that although the extent of alignment did not differ between the ostracism and control groups, there may have been different mechanisms underpinning alignment behaviour in the two groups: Language ability (i.e., receptive vocabulary) contributed significantly to alignment effects of children in the control group, whereas social skill contributed significantly to alignment effects of children in the ostracism group.

### Bayesian analysis

Our Bayesian analysis quantified the likelihood that we would observe our data if there were no difference in the alignment effects between the ostracism and control groups, compared to if there were a difference between the groups (Wagenmakers, 2007). To carry out this analysis, we fitted a null LMEM to our snap! game data, which excluded the fixed effect of group; this LMEM assumed that there was no difference in the extent of alignment between the ostracism and control groups. We compared the null LMEM to the alternative (full) LMEM described in Table 3, which—by including the fixed effect of group—assumed that the two groups might differ in the extent of their alignment. We then used the BIC values of the null and alternative LMEMs to calculate a Bayes Factor as e^(BIC_alternative − BIC_null)/2^ (see Masson, 2011).

The null LMEM (i.e., without the effect of group) fit the snap! game data better by a Bayes Factor of e^(497.29 − 472.24)/2^ = 274,134.10, offering strong evidence against the hypothesis that children in the ostracism and control groups differed in the magnitude of their alignment (posterior probability in favor of the null model BF / (BF + 1) = 0.99, which is very strong evidence according to Raftery’s categorization; Raftery, 1995). This test provides additional confidence that our findings are not just a function of a small sample size.

## Discussion

Autism is associated with clinically significant impairments of communication and social interaction (DSM-5; American Psychiatric Association, 2013), which manifest in conversation (Ying Sng et al., 2018). Typical adults and children imitate a conversational partner’s language choices (Brennan & Clark, 1996; Giles et al., 1991; Pickering & Garrod, 2004) and such alignment promotes effective, rewarding exchanges between speakers (Fusaroli et al., 2012; Putman & Street, 1984). The current study investigated whether autistic children’s priming-based tendency to align (Allen et al., 2011; Branigan et al., 2016; Hopkins et al., 2017) can be socially modulated, in a way that might promote affiliation within dialogue. Consistent with previous research, we showed that autistic children display spontaneous lexical alignment with an experimenter (Branigan et al., 2016; Hopkins et al., 2017). Yet we found no evidence that they modify their linguistic behavior to achieve affiliative goals: Unlike their typically-developing peers (Hopkins & Branigan, 2020), autistic children who had experienced ostracism did not display increased lexical alignment, relative to controls. This finding suggests that linguistic imitation is unresponsive to social manipulation in autism, in the same way as is non-linguistic imitation (Cook & Bird, 2012).

However, it is argued elsewhere (Slocombe et al., 2013) that we should not assume that the same mechanisms underpin linguistic alignment in two groups, just because the groups show the same level of alignment. This observation seems relevant to the current study: Although the behavioural results of the ostracism and control groups were the same, our analyses suggested group-specific patterns in our data. The patterns are consistent with Branigan et al.’s (2010) proposal that observed alignment behaviors may arise as the outcome of multiple underlying mechanisms. Specifically, although there was a negative relationship between alignment effects and SCQ scores for all children (i.e., reduced lexical alignment was associated with increased autism symptom severity), this relationship was especially strong for the ostracism group. This suggests that, when social-affective mechanisms could be expected to be relevant to alignment, they were—albeit not to the extent that they yielded observable changes in alignment behavior (i.e., significantly stronger alignment effects). Furthermore, there was a significant interaction between prime name and SCQ scores for children in the control group only. This suggests that, in circumstances where there was no specific pressure to affiliate, automatic psycholinguistic mechanisms (i.e., priming) seem to have been more relevant to autistic children’s lexical alignment. Overall, then, our results imply that our experimental manipulation may have engaged different alignment mechanisms in the ostracism and control groups, but not sufficiently to elicit the reliable differences in alignment behaviors observed in typically-developing children (Hopkins & Branigan, 2020). However, since the inclusion and ostracism groups were small in our study, observations of a larger sample would be important in corroborating the finding of reduced affiliative influence on ostracised autistic children’s tendency to align, and the possibility that different mechanisms might differentially contribute to their alignment behaviours.

Hence the question arises: Given that ostracism influences the lexical alignment of typically-developing children (Hopkins & Branigan, 2020), why did we not observe the same effects in an autistic sample? An obvious explanation would be that our sample size was too small to detect such an effect. However, the results of our Bayesian analysis, which provides a measure of the strength of evidence for one hypothesis over another, casts doubt on this possibility. Despite our relatively small sample size, the Bayes Factor quantifying the strength of evidence in favor of the null hypothesis was substantial, which provided very strong evidence against the hypothesis that autistic children’s inclusionary status influenced the extent of their alignment (i.e., the alternative hypothesis; Raftery, 1995).

Another obvious explanation would be that our ostracism manipulation—Cyberball (Williams et al., 2000)—was ineffective. Again, we suggest that this is unlikely to have been the case, since our manipulation check scores suggested that children experienced Cyberball as intended; that is, children in the ostracism group appropriately reported receiving the ball significantly less than did children in the control group.

This leaves two alternative explanations of our findings, which may not be mutually exclusive. One possibility is that children’s manipulation check scores reflected their experience of inclusion/ostracism but failed to capture how this experience affected them emotionally. We intentionally avoided having a manipulation check that (i.) required introspection (an area of difficulty for autistic children; Robinson et al., 2017) and (ii.) explicitly drew attention to the purpose of the manipulation—unlike other studies that have used Cyberball with an autistic population (e.g., Sebastian et al., 2009, who asked participants to rate statements such as “I was excluded”). However, our choices meant that we were unable to determine that children’s assessment of how often they received the ball corresponded with their feelings about being included/ostracised by the confederate avatars. It is plausible that children did not feel included/ostracised in the way (and/or to the extent) that we had anticipated, despite their responses on the manipulation check. Such an explanation would be consistent with evidence of discrepancies between how autistic people process versus how they report experiences of ostracism (Bolling et al., 2011; Masten et al., 2011; McPartland et al., 2011; Sebastian et al., 2009; Trimmer et al., 2017).

A closely related possibility is that although the experience of ostracism might have been emotionally significant for autistic children, this might not have increased their motivation to affiliate with others; such a pattern would be consistent with other findings on social motivation in autism (Chevallier, Grèzes, et al., 2012; Chevallier, Kohls, et al., 2012). In turn, under a goal-activation account of affiliation (Aarts & Dijksterhuis, 2000), any reduced motivation to fulfil affiliative goals in autistic children would have meant that ostracised autistic children would not have been more likely to engage in affiliative behaviours, like their ostracised typically-developing peers (Hopkins & Branigan, 2020; Over & Carpenter, 2009; Song et al., 2015). This explanation would be coherent with evidence for atypical social modulation of non-linguistic imitation in autism (Wang & Hamilton, 2012; Vivanti & Hamilton, 2014; Marsh et al., 2013; Vivanti & Dissanayake, 2014; Vivanti et al., 2016; Cook & Bird, 2012), and with studies that show that autistic children can recognise but not always act to rectify their social isolation (Bauminger & Kasari, 2000).

Hence we have proposed different but complementary accounts of how autistic children’s experience of ostracism might preclude knock-on effects on lexical alignment. We assume that, if autistic children did not feel the negative effects of ostracism, and/or were not motivated to deploy behavioral strategies (e.g., imitation) to compensate for these negative effects, then they would not have displayed alignment above the level generated by automatic priming mechanisms (Branigan et al., 2016; Hopkins & Branigan, 2020; Hopkins et al., 2017). Future research in this area should establish what—for autistic children—disrupts the relationship between ostracism and increased affiliative behaviour that is seen in the typical population (Carpenter et al., 2013; Dijksterhuis & Bargh, 2001). For example, one way of addressing the potential gap between autistic children’s experience versus their reporting of ostracism would be to take additional measures during Cyberball—both physiological and psychological—as per Trimmer et al. (2017), which could help to determine whether the absence of heightened alignment effects reflects atypical emotional processing or something else.

The lack of difference in alignment behaviors between the groups in our study carries practical implications for autistic children and their social partners. Our findings suggest that a potentially valuable form of behavioral adaptation is unavailable to autistic children, which could offer a new perspective on why their conversational behavior appears unusual (Ying Sng et al., 2018). Furthermore, given the bi-directional link between imitation and affiliation in typical speakers (Dijksterhuis & Bargh, 2001), the reduced sensitivity of autistic children’s language imitation to ostracism—and potentially social affiliative considerations more generally—could result in their conversational partners experiencing interactions with them as unrewarding, and hence result in a reduced motivation for these partners to engage in further interaction with them. This could further entrench the social difficulties faced by autistic children, which include poorer quality friendships and increased feelings of loneliness (Bauminger & Kasari, 2000); social isolation at school (Kasari et al., 2011) and even strained familial relationships (Kaminsky & Dewey, 2001).

We conclude by suggesting that the social-affective mechanisms contributing to alignment are impaired for autistic children. In the present study, autistic children imitated the lexical choices of a partner, but those who experienced ostracism were as likely to imitate lexical choices as those who experienced inclusion; this is a different pattern to that found in typically-developing children, who show a stronger tendency to lexically align when they have been ostracised (Hopkins & Branigan, 2020). Intriguingly, however, our study offers evidence that autistic children’s language alignment might be underpinned by different mechanisms in different contexts; we showed that linguistic ability predicted children’s alignment in the control group, whereas social-communicative skills were predictive in the ostracism group. Such findings imply that, for autistic children, alignment mechanisms may be engaged in a way that is selective and contextually appropriate, but beneath a threshold to induce noticeable modulation of alignment behaviour.
